# Current Evidence and Future Perspectives in the Medical Management of Vascular Ehlers–Danlos Syndrome: Focus on Vascular Prevention

**DOI:** 10.3390/jcm13144255

**Published:** 2024-07-21

**Authors:** Giacomo Buso, Federica Corvini, Elena Maria Fusco, Massimiliano Messina, Fabio Cherubini, Nicola Laera, Anna Paini, Massimo Salvetti, Carolina De Ciuceis, Marco Ritelli, Marina Venturini, Nicola Chiarelli, Marina Colombi, Maria Lorenza Muiesan

**Affiliations:** 1 Department of Clinical and Experimental Sciences, Division of Internal Medicine, ASST Spedali Civili Brescia, University of Brescia, 25123 Brescia, Italy; 2 Faculty of Biology and Medicine, University of Lausanne, 1015 Lausanne, Switzerland; 3 Department of Molecular and Translational Medicine, Division of Biology and Genetics, University of Brescia, 25123 Brescia, Italynicola.chiarelli@unibs.it (N.C.);; 4 Department of Clinical and Experimental Sciences, Division of Dermatology, ASST Spedali Civili Brescia, University of Brescia, 25123 Brescia, Italy

**Keywords:** vascular Ehlers–Danlos syndrome, pharmacological treatment, celiprolol, vascular prevention, gene therapy, non-coding RNA

## Abstract

Vascular Ehlers–Danlos syndrome (vEDS) is a rare autosomal dominant connective tissue disease resulting from pathogenic variants in the collagen type III alpha 1 chain (*COL3A1*) gene, encoding type III procollagen. Patients with vEDS present with severe tissue fragility that can result in arterial aneurysm, dissection, or rupture, especially of medium-caliber vessels. Although early reports have indicated a very high mortality rate in affected patients, with an estimated median survival of around 50 years, recent times have seen a remarkable improvement in outcomes in this population. This shift could be related to greater awareness of the disease among patients and physicians, with improved management both in terms of follow-up and treatment of complications. Increasing use of drugs acting on the cardiovascular system may also have contributed to this improvement. In particular, celiprolol, a β1 cardio-selective blocker with a β2-agonist vasodilator effect, has been shown to reduce rates of vascular events in patients with vEDS. However, the evidence on the true benefits and possible mechanisms responsible for the protective effect of celiprolol in this specific setting remains limited. Drugs targeting the extracellular matrix organization and autophagy–lysosome pathways are currently under investigation and could play a role in the future. This narrative review aims to summarize current evidence and future perspectives on vEDS medical treatment, with a specific focus on vascular prevention.

## 1. Introduction

Vascular Ehlers–Danlos Syndrome (vEDS, MIM#130050) is a rare autosomal dominant heritable connective tissue disorder with an estimated prevalence of 1/150,000 [1]. It is caused by mutations in the collagen type III alpha 1 chain (*COL3A1*) gene, which encodes the pro-alfa1 chain of type III procollagen, a fibrillary protein mainly expressed in the walls of blood vessels and hollow organs. The resulting tissue fragility increases the risk of arterial rupture, dissection, and aneurysm development, as well as rupture of the bowel and gravid uterus. Spontaneous pneumothorax, congenital hip dislocation, and *talipes equinovarus* can also occur. Patients may also display typical physical features such as thin nose and lips, hollow cheeks, prominent eyes, lobeless ears, translucent skin with prominent vascular pattern, acrogeria, easy bruising, atrophic scars, as well as small joint hypermobility. The diagnosis is suspected in the presence of the above clinical features or in family members of affected patients, though disease confirmation needs molecular genetic testing [2]. Patients with vEDS exhibit high morbidity and mortality mainly from vascular events. Historically, the median life expectancy has been estimated to be around 50 years [3,4], with arterial complication rates around 1.3 events/5 years and overall organ complication rates reaching 1.6 events/5 years [5]. Major complications occur in up to 85% of cases by the age of 43 [1]. For instance, endovascular and surgical treatment outcomes are poor in this setting with complication rates as high as 46%, so prevention is essential [6,7]. In 2010, the Beta Blocker in Ehlers–Danlos Syndrome Trial (BBEST) demonstrated a 64% reduction in the risk of arterial rupture or dissection in patients treated with celiprolol compared with controls [5]. After this first attempt, no other medication has been clearly proven effective in preventing vascular complications in patients with vEDS, though ongoing trials are investigating other cardiovascular drugs, such as angiotensin receptor blockers (ARBs), which have yielded positive results in mouse models [8]. The urgent need for new treatment strategies is emphasized by the fact that patients with vEDS still experience excessively high rates of serious vascular complications despite celiprolol therapy, as shown recently [9].

**Figure 1:**
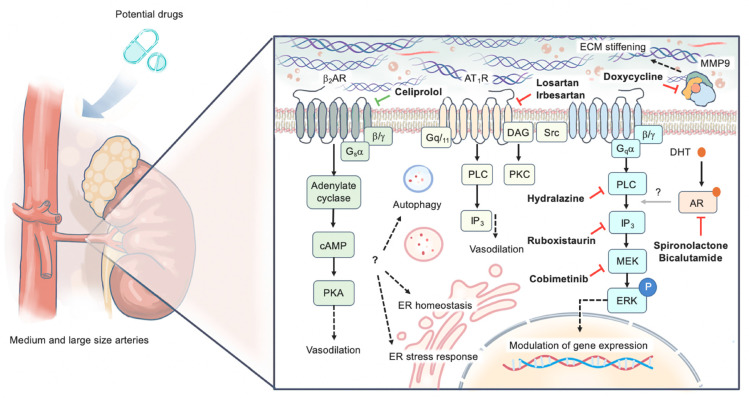
Potential therapeutic approaches to vascular Ehlers–Danlos syndrome. Celiprolol may reduce total peripheral resistance by dilating skeletal muscle resistance vessels through β2-adrenergic receptors and might decrease arterial wall shear stress by lowering heart rate through β1-adrenergic receptor antagonism. This drug could also reduce vascular oxidative stress at the arterial wall level by stimulating nitric oxide production. Lastly, it might increase arterial stiffness by enhancing TGFβ-mediated pro-fibrotic mechanisms and promoting collagen synthesis via β1 antagonism and β2 stimulation, respectively. By acting on angiotensin receptors, sartans could provide vascular protection either by inducing vasodilation or by limiting the adverse effects of angiotensin II on vascular remodeling. Targeting matrix metalloproteinases (MMPs) via doxycycline might counteract collagen degradation, thereby increasing its content in the arterial wall and enhancing vessel stiffness. Inhibition of phospholipase C/inositol 1,4,5-triphosphate/protein kinase C/extracellular signal-regulated kinase (PLC/IP3/PKC/ERK) has been shown to prevent spontaneous fatal aortic rupture in a mouse model. Antiandrogen medications may also contribute by interfering with this signaling pathway. Future research should identify drugs capable of improving mechanisms of autophagy, endoplasmic reticulum (ER) homeostasis, and stress response, which are likely to contribute substantially in the natural history of vEDS. Red lines: inhibition; green lines: stimulation; AR: androgen receptor; AT_1_R: angiotensin 1 receptor; β_2_AR: β2-adrenergic receptor; DHT: dihydrotestosterone; ECM: extracellular matrix; ER: endoplasmic reticulum; MMP: matrix metalloproteinase.

The research has advanced greatly in recent years to overcome this gap, but there is still a long way to go. The aim of this narrative review is to summarize the current evidence on medical management of vEDS and provide future perspectives on possible novel treatment strategies (depicted in Figure 1), with a specific focus on vascular prevention.

## 2. Natural History of Vascular Ehlers–Danlos Syndrome: What Has Changed?

It is widely acknowledged that vEDS is an ominous condition even in young individuals. A study published in 2000 by Pepin et al. showed a median survival of 48 years, with over 80% of the patients experiencing at least one complication by the age of 40 [3]. These data were supported by a subsequent work on more than 1200 patients, where median survival was particularly low in the male sex, mainly due to higher rates of death from vascular rupture by the age of 20 than in female counterparts [4].

Impressively enough, the prognosis of patients with vEDS seems to have markedly improved over the last 20 years. An observational study by Frank et al. including 144 affected subjects followed in the French National Referral Center for Rare Vascular Diseases between 2000 and 2017 found an overall survival rate of 71.6% during a 5.3-year follow-up period and over 80% by the age of 50 [10]. Comparable results were obtained in a recent multicenter observational study from the UK, where the 5-year survival was estimated around 90% [11].

Several factors may explain this shift in the natural history of vEDS. First, vEDS management has significantly improved both in terms of the prevention and management of complications. In fact, the creation of dedicated, specialized centers in several countries has led to easier access to care while promoting patient education. Furthermore, several centers have implemented protocols to enhance vascular surveillance through regular follow-up imaging [10,11]. Another relevant point is pharmacological therapy. In the above study by Frank et al., for example, about 90% of the patients were on celiprolol therapy. These subjects displayed significantly improved survival than those with no such treatment during the study period (72.4% vs. 52.2%, respectively; *p* < 0.001) [10]. Similarly, in the study by Bowen et al., survival at 5 years was 96.53% for those on any cardiovascular medication (including other β-blockers and ARBs) and 42.69% for patients in the control group [11]. Taken together, these data suggest that the systematic use of β-blockers and/or inhibitors of the renin–angiotensin–aldosterone system (RAAS) may have played a crucial role in improving outcomes of patients with vEDS in recent times. However, evidence supporting this hypothesis remains scarce, as described below.

## 3. Celiprolol: Cornerstone Treatment but Still Debated

### 3.1. The BBEST Study

Celiprolol is a third-generation β-blocker with β1-adrenoceptor antagonism and partial β2-agonism. In normotensive people, the β2-agonist properties predominate [12]. Because of such unique characteristics, this drug may be better described as a selective adrenoreceptor modulator [13,14]. Like other β-blockers, celiprolol has both antihypertensive and antianginal activity. However, the drug lacks the typical side effects of β-blockers, such as bronchoconstriction and peripheral vasoconstriction [14]. Its blockade of β1-adrenoreceptors reduces the sympatho-adrenergic stimulation of the heart, decreasing the heart rate and myocardium contractility [15]. Celiprolol also stimulates β2-receptors, improving vasodilatation [16,17,18]. As evidenced by a 2013 study of porcine coronary arteries, celiprolol may also act as a β3-adrenoceptor agonist with resulting vasorelaxation [19]. Additionally, it may induce coronary vasodilation by inducing the release of nitric oxide [20].

In the past century, no specific medical treatment was available for patients with vEDS. In the late 90s, a group of French specialists hypothesized that a drug capable of reducing arterial wall stress while avoiding excessive vasodilatation and heart rate reduction might have improved the outcome in patients with vEDS. Celiprolol was chosen due to its unique properties. In fact, true vasodilators were unlikely to be well tolerated in subjects with vEDS, as they often have normal or even low blood pressure levels. Classical non-selective or selective β1-antagonsits were discarded as well to avoid the risk of chronic fatigue related to reduced heart rate in otherwise young and active patients [12]. These considerations led to the BBEST, the first randomized trial on the effects of celiprolol in preventing cardiovascular events in the setting of vEDS. In this study, published in 2010, 53 patients were randomly assigned to treatment either with celiprolol with a target dose of 400 mg daily or with usual treatment and no β-blocker. The mean duration of follow-up was 47 months. The trial was stopped early for benefit, as celiprolol led to a threefold reduction in arterial ruptures or dissections in the treatment arm (hazard ratio 0.36; 95% confidence interval 0.15–0.88; *p* = 0.040) [5]. Despite such encouraging results, this study has some important limitations worth mentioning and was therefore widely criticized afterwards [5,12], as discussed in the dedicated section below.

### 3.2. Evidence from Experimental Models

Interestingly, a recent experimental study by Dubacher et al. in a mouse model of vEDS suggested that celiprolol but not losartan can improve the biomechanical integrity of the aortic wall, thereby potentially reducing the risk of dissection and rupture. Doxycycline, a tetracycline antibiotic capable of inhibiting MMPs, also positively impacted the mechanical properties of the aorta in treated mice [21]. Targeting the balance of collagen homeostasis through inhibition of such enzymes had already proven beneficial in earlier preclinical studies on *Col3a1* haplo-insufficient mouse models [22,23]. However, offering doxycycline in the long term to patients with vEDS is unrealistic.

In addition to the classical β-blocker action, celiprolol stimulates nitric oxide production and thus may reduce vascular oxidative stress at the arterial wall level. The link between β adrenergic stimulation and TGFβ expression is another potential mechanism to mention in this regard [24]. In fact, celiprolol may enhance TGFβ-mediated pro-fibrotic mechanisms through β1 antagonism while promoting collagen synthesis via β2 stimulation [13]. This drug could therefore have a protective effect by increasing arterial stiffness. Accordingly, Boutouyrie et al. demonstrated significantly higher carotid distensibility and circumferential and pulsatile wall stress in patients with vEDS than in healthy controls [25], whereas celiprolol selectively increased Young’s elastic modulus by reducing carotid distensibility among BBEST participants [5]. Importantly, a later study using another mouse model failed to demonstrate such a protective effect of celiprolol [8], whereas another work even suggested that the drug could accelerate rather than reduce death from aortic rupture in two different mouse models [26]. Therefore, the precise effects of celiprolol in animal models of vEDS remain to be elucidated.

In a recent case report published by Ishikawa et al., a skin biopsy was performed before and after 3 years of celiprolol administration in a patient with vEDS. Although the expression level of procollagen III did not change, the size differences in the collagen fibrils improved. Furthermore, the ER dilatation observed in skin fibroblasts before treatment, which indicated ER stress, was no longer noticed [27]. Such preliminary data suggest an additional potential protective effect of celiprolol in patients with vEDS through these mechanisms as well, though further research should confirm this hypothesis.

Key findings from experimental models using celiprolol and other pharmacological agents in the setting of vEDS are summarized in Table 1.

### 3.3. Further Clinical Evidence

From a clinical perspective, after the BBEST, several observational studies have supported the protective role of celiprolol in vEDS. In 2019, Frank et al. published a retrospective study describing the outcomes of 144 molecularly confirmed vEDS patients followed in a French referral center between 2000 and 2017. All patients were recommended treatment with celiprolol (≤400 mg/day) on top of usual care. After a median follow-up of 5.3 years, overall patient survival rate was 71.6%, which was dependent on the *COL3A1* variant, age at diagnosis, and medical treatment. In particular, patients treated with celiprolol had a better survival rate than controls (*p* = 0.0004) and the observed reduction in mortality was dose-dependent, with the best protection being provided at the dose of 400 mg daily compared with lower doses (*p* = 0.003) [10]. In another study published in 2021 by a Swedish group, 40 patients with molecularly confirmed vEDS were offered celiprolol treatment in the period between 2011 and 2019. With a median follow up of 22 months, the annual risk of a major vascular event was 4.7%, which was comparable to that obtained in patients receiving celiprolol in the BBEST (5%) and lower than in untreated patients from the same study (12%) [28]. Recently, Buso et al. published their personal experience with celiprolol in a cohort of 26 patients with molecularly confirmed vEDS followed at the Brescia University Hospital, Italy, from 2011 to 2023. At the last follow-up visit, all patients were on celiprolol therapy, 80% of whom were taking the maximum recommended dose of 400 mg daily. Whilst the drug was well tolerated overall, the yearly risk of symptomatic vascular events was still not negligible (8.8%), whereas the majority of the events occurred after reaching the maximum recommended dose of celiprolol [9].

In light of these data, the insufficient body of evidence supporting the efficacy and safety of celiprolol both in animal models and patients with vEDS, and the many limitations of the studies published so far, celiprolol is still not approved by the Food and Drug Administration (FDA) and is therefore not available for the treatment of vEDS in the US. A prospective, phase 3, randomized, double-blind, placebo-controlled efficacy study (ClinicalTrials.gov Identifier: NCT05432466) is ongoing and will hopefully provide some more robust data by the end of 2025.

The clinical evidence on celiprolol and further potential drugs in the setting of vEDS is reported in Table 2.

## 4. Is Any Drug Better than None? Potential Role of Angiotensin II Receptor Antagonists

As celiprolol is not available in several countries and after the publication of the BBEST study and the experimental paper by Dubacher et al. [21], the question arose whether additional drugs acting on the cardiovascular system would elicit the same protection in the setting of vEDS. In a study using a *Col3a1*^m1Lsmi^ mouse model, Gorosabel et al. found that bisoprolol, a β1-selective antagonist, did not improve the biomechanical integrity of the thoracic aorta of treated mice compared with controls, suggesting that the beneficial effect of celiprolol may not be extrapolated to bisoprolol in humans [29].

In addition to the regulation of β-adrenergic receptor function, several preclinical studies have highlighted the potential role of angiotensin II antagonism in vascular protection in the setting of vEDS. In 2013, Faugeroux et al. showed that angiotensin II infusion increased systolic blood pressure in both *Col3a1^+/−^* and *Col3a1^+/+^* mice but led to a significantly higher rate of premature mortality in the former, particularly during the first week of infusion (55% vs. 0%). Remarkably, reducing angiotensin II doses significantly reduced mortality rates in this group. The authors therefore concluded that *Col3a1* haplo-insufficient mice exhibit increased susceptibility to develop premature thoracic aortic rupture in response to angiotensin II and the associated increase in blood pressure levels [30]. In a more recent French study on knock-in *Col3a1*^+/G182R^ mice leading to glycine substitution (the most common mutation observed in patients with vEDS), different antihypertensive therapies, alone or in combination, were compared in terms of effects on survival within the first 24 weeks of life [8]. The drugs used in this study targeted heart rate and/or systolic blood pressure to decrease wall stress. In particular, mice were treated with different β-blockers such as propranolol and celiprolol, and the dihydropyridine calcium channel blockers amlodipine, losartan, and hydralazine. Among these medications, the only one that significantly reduced the risk of death from spontaneous rupture of the thoracic aorta was losartan. Of note, the discontinuation of losartan induced a return to increased mortality from aortic rupture similar to that in untreated mice, whereas initiation of losartan after weaning had the same strong beneficial effect on survival. Furthermore, administration of angiotensin II at doses with minimal effects on systolic blood pressure induced aortic rupture with a 100% mortality rate within 10 days after administration, suggesting that the deleterious effect of angiotensin II in vEDS may be at least partially unrelated to the increased blood pressure levels [8]. In this regard, it is noteworthy that angiotensin II induces vascular remodeling through smooth muscle cell hypertrophy and secretion of extracellular matrix (ECM) proteins via multiple canonical and noncanonical signaling pathways [31]. If altered, such mechanisms could contribute to aortic fragility in individuals with vEDS [8,30]. Moreover, angiotensin 1 receptors are known to modulate sympathetic vasomotor function [32], which could also play a deleterious role in this setting. All these mechanisms could also be targeted by ARBs.

From a clinical perspective, a recent retrospective study on 126 patients with molecularly confirmed vEDS followed in two referral centers of the UK EDS National Diagnostic Service [11] showed that, regardless of the cardiovascular drug used, patients treated with celiprolol, other β-blockers, and/or ARBs, alone or in combination, had a significantly more favorable outcome than untreated subjects (96.53% vs. 42.67%, respectively, at 5 years). However, the small sample size, lack of randomization, and the potential presence of confounding factors are major limitations to consider. The Angiotensin II Receptor Blockade in Vascular Ehlers–Danlos Syndrome (ARCADE) study, a multicenter, double-blind, randomized, placebo-controlled, parallel group study with blind endpoint evaluation investigating the efficacy and safety of irbesartan in adult patients with vEDS (ClinicalTrials.gov Identifier: NCT02597361), has been completed and hopefully its results will be announced soon.

## 5. Role of the PLC/IP3/PKC/ERK Signaling Pathway: New Hope or Nope?

Recently, dysregulation of the PLC/IP3/PKC/ERK signaling pathway emerged as a potential contributor to vEDS pathogenesis, as well as another promising therapeutic target in the near future. In physiological conditions, this complex intracellular signaling cascade is involved in various cellular processes, including cell growth, differentiation, and survival [33]. Using two heterozygous *Col3a1* mutations-carrying mouse models of vEDS (*Col3a1*^G209S/+^ and *Col3a1*^G938D/+^), Bowen et al. [26] demonstrated that abnormalities in this signaling pathway are likely involved in spontaneous vascular events in the setting of vEDS, as pharmacologic inhibition of PKCβ or ERK1/2 led to better outcomes in mutated mice by preventing spontaneous fatal aortic rupture. In particular, mice treated with ruboxistaurin, a PKCβ inhibitor, displayed increased survival after 45 days compared with controls (94% vs. 52%, respectively), suggesting that PKC-dependent ERK activation may be a critical driver of aortic disease in vEDS. Similarly, the administration of cobimetinib, a specific inhibitor of MEK, resulted in 90% survival after 45 days. Intriguingly, hydralazine improved survival in treated mice when initiated at birth, though protection from aortic rupture was abruptly lost around the time of sexual maturity, particularly in males. Hydralazine is an anti-hypertensive medication that acts, at least partially, by blocking the IP3-mediated calcium release from the ER and hence PKCβ activation. The above finding suggests that androgens could influence the signaling pathway by acting distally to IP3. In fact, concomitant treatment with androgen receptor antagonist bicalutamide resulted in increased survival in both sexes. Similar results were found in mice treated with both hydralazine and spironolactone [26].

Although a subsequent study using another knock-in mouse model (*Col3a1*^+/G182R^) did not confirm these aspects [8], the above findings generated great enthusiasm in the scientific community, as they shed light after a long time on the pathophysiological mechanisms of vEDS and for the potential therapeutic repercussions. In fact, shortly after the study was published, the Prevention of Rupture with Enzastaurin in Vascular Ehlers–Danlos Syndrome (PREVEnt) trial was announced (ClinicalTrials.gov Identifier: NCT05463679). This two-arm, multicenter, randomized, double-blind, placebo-controlled study aimed at evaluating the efficacy and safety of AR101 (enzastaurin) 500 mg once daily compared to placebo in preventing arterial events in individuals with vEDS. Enzastaurin is another PKCβ/PI3K/AKT signaling inhibitor that has shown promising results in patients with brain tumors and is commonly used in clinical practice [34]. Unfortunately, in October 2022, the company sponsoring the PREVEnt trial announced the indefinite suspension of the study for commercial reasons [35], and we still do not know whether and when this avenue will be further explored in the future.

## 6. Gene Therapy: A Road Yet to Be Taken

Current treatments for vEDS are only symptomatic due to our limited understanding of its pathomechanisms. In-depth molecular and cellular studies are essential to identify therapeutic targets. Beyond pharmacological approaches, gene therapy strategies hold the potential to cure the disease by addressing its root cause.

Concerning the potential of gene therapy for treating vEDS, RNA interference (RNAi) technology has successfully targeted the expression of the mutant *COL3A1* allele harboring a glycine substitution (p.Gly252Val) in patients’ dermal fibroblasts. This approach effectively targets the mutant mRNA while preserving the wild-type allele. Moreover, it has been associated with enhanced collagen fibril formation and reduced negative effects of unfolded proteins, suggesting promising therapeutic potential [36].

In addition to RNAi, RNA editing represents an even more promising RNA-targeting strategy, even if the efficient delivery of RNA editing components to specific tissues, especially the vasculature, remains a significant challenge. RNA editing, particularly A-to-I editing, offers the potential to therapeutically correct pathogenic single nucleotide variants in the human transcriptome with minimal risk of creating permanent off-target edits in the genome, unlike some other editing techniques [37]. A-to-I editing involves the hydrolytic deamination of adenosine (A) to inosine (I) and is facilitated by adenosine deaminases acting on RNA enzymes. Given that G-to-A mutations account for nearly half of all known disease-causing point mutations in humans [38] and many clinically relevant mutations in *COL3A1* involve pathogenic G-to-A mutations causing glycine substitutions [39], A-to-I conversion by RNA-editing systems presents significant therapeutic potential. This approach could correct disease-causing mutations, offering immense opportunities for developing targeted treatments for vEDS patients.

## 7. Recent Evidence on Non-Coding RNAs as Molecular Targets

Epigenomic studies focusing on the modulation of non-coding RNA (ncRNAs) expression in cardiovascular conditions, including thoracic/abdominal aortic aneurysms and vascular connective tissue disorders like Loeys–Dietz and Marfan syndromes, have provided valuable insights into the dysregulated gene regulatory pathways linked to the underlying disease mechanisms. These research efforts have elucidated how ncRNAs, especially microRNAs (miRNAs), may influence the expression of key genes involved in maintaining vascular health and contributing to their pathogenesis [40].

To better understand the complex vEDS pathomechanisms and identify potential molecular targets for therapeutic intervention, a recent study by Chiarelli et al. [41] employed an integrative multi-omics approach on patient-derived dermal fibroblasts. This comprehensive transcriptomics and miRNomics analysis provided an in-depth view of the molecular alterations in vEDS. It revealed a complex pathological interplay between disrupted ECM organization, altered ER proteostasis, and defective autophagy. The detailed miRNA expression profile of patient fibroblasts offered the first disease-specific signature, underscoring the critical role of miRNAs in the vEDS pathobiology. Specifically, epigenetic changes caused by the aberrant expression of several miRNAs, including miR-15b-5p, miR-16-5p, miR-21-3p, miR-24b-3p, miR-29a-3p, miR-29b-3p, miR-138-5p, miR-145-5p, and miR-195-5p, can affect the dysregulated gene expression networks by regulating transcription of a range of targets. Among them, the upregulation of miR-29 family members, i.e., miR-29a-3p and miR-29b-3p, appears to significantly disrupt ECM turnover and cell survival processes, such as ER proteostasis and the autophagy–lysosome pathway, essential for maintaining vascular integrity. Notably, the increased expression of miR-29a/b is strongly associated with the downregulation of several genes involved in vascular ECM structure and turnover (e.g., collagens, elastin, ECM-modifying enzymes), contributing to aortic damage and aneurysm development [42]. These miRNAs could emerge as critical molecular targets for vEDS, impacting the vascular pathogenesis. Ongoing research targeting miR-29a and miR-29b holds promise for developing tailored therapies addressing the condition’s burden and improving patient management.

## 8. Limits of Available Evidence and Challenges to Overcome

Several aspects diminish the strength of the above studies and need to be mentioned. First, although celiprolol is the only drug tested in a clinical trial, evidence supporting its efficacy and safety in patients with vEDS remains very limited. In the BBEST study, investigators were not blinded with respect to the treatment, since creating a placebo with the same aspect as celiprolol tablet was excessively expensive [12]. The small number of patients recruited and the fact that only 33 out of 53 participants had proven *COL3A1* mutations constitute additional limitations worth mentioning [5]. On the other hand, the remaining clinical evidence supporting the use of celiprolol in vEDS stems from observational studies, not designed for adequate control with placebo or further treatments [9,10,28].

Discrepancies between the results obtained by using celiprolol and other drugs in various mouse models is also noteworthy. The first models generated harbored heterozygous or homozygous null mutations, which do not capture the full spectrum of mutations responsible for the most common vEDS subtypes, like glycine substitutions or exon skips [43]. Later, more advanced and reliable models carrying knock-in mutations similar to those known to induce vEDS in humans have partially overcome such pitfalls [8,21,26,29]; however, conflicting evidence still imposes caution in the interpretation of preclinical studies in this and other conditions, such as Marfan syndrome [44].

While RNAi-based gene therapy and RNA editing show promise for vEDS, several challenges must be overcome to make these viable treatment options. Developing allelic-specific knockdown for diverse array of *COL3A1* alleles is essential for ensuring precision and effectiveness. Additionally, achieving the efficient delivery of RNAi and RNA editing to treat tissues, particularly the vasculature, is crucial. The dynamic environment of the vascular system, characterized by constant movement and complex architecture, complicates the development of delivery mechanisms that can achieve precise targeting without causing adverse effects. Ensuring the long-term expression of therapeutic agents is another significant hurdle. This requires developing systems that can maintain stable and consistent gene expression over extended periods, avoiding the degradation or inactivation of therapeutic RNA. Furthermore, preventing immune responses to these therapeutic agents is critical, as the body’s natural defense mechanisms could potentially neutralize the therapy or cause harmful side effects. The specificity of RNAi technologies also presents a challenge. Off-target effects, where the therapy inadvertently affects unintended genes or cellular processes, can lead to unexpected consequences, making it vital to enhance the precision of these technologies.

The timeline for gene therapies to become available for vEDS patients is contingent on overcoming the above-mentioned challenges and navigating clinical trials. Typically, the development and approval process for new gene therapies can span a decade or more, involving preclinical studies, multiple phases of clinical trials, and regulatory review. Given the current pace of research and advancements in RNA technologies, it is conceivable that effective gene therapies for vEDS could become available within the next 10 years or less. For instance, the RNAi therapeutic agent Patisiran, used to treat hereditary transthyretin amyloidosis, has shown remarkable success and received FDA approval within a relatively short period [45]. Regarding RNA editing, Wave Life Sciences advanced the first-ever adenosine deaminases acting on RNA (ADAR)-based RNA editor into healthy volunteers in 2023 for alpha-1 antitrypsin deficiency, with plans to treat patients soon. In parallel, a growing number of biotechs are setting their sights on similar RNA-editing opportunities for various monogenic disorders, including cardiovascular diseases [46]. These precedents suggest that with continued research, the development of gene therapies for vEDS is feasible, albeit with its unique set of challenges.

## 9. Practical Implications: How to Protect Patients with Vascular Ehlers–Danlos Syndrome in 2024?

In light of the aforementioned, one may question how the currently available evidence translates into clinical practice. Owing to the congenital, chronic, and aggressive nature of vEDS, the best preventative strategy in affected patients is likely to be a multidisciplinary approach in referral centers, so as to ensure adequate care and long-term follow-up [10]. Drug therapy may also play a key role in disease management, though treatment options remain very limited in this setting, as extensively discussed above.

In our Center for Rare Vascular Diseases at the University Hospital of Brescia (“Spedali Civili di Brescia”), Italy, individuals referred with vEDS are monitored and treated according to our local clinical protocols (“Percorsi Diagnostico Terapeutici Assistenziali”), which basically reflect the existing evidence. Once the presence of a causative mutation is confirmed, patients undergo a series of instrumental investigations to assess in particular the status of their cardiovascular system, including thorough clinical evaluation, office blood pressure measurement and 24 h ambulatory blood pressure monitoring, transthoracic echocardiography, as well as whole-body vascular imaging with computed tomography or magnetic resonance angiography. Unless contraindicated, celiprolol therapy is offered to all patients, as this remains the only drug tested so far in a clinical trial in this setting. Follow-up is generally proposed every 12 to 18 months, except in cases of intercurrent clinical conditions necessitating more closer monitoring. Celiprolol is titrated progressively up to a maximum of 400 mg per day, based on patients’ age and comorbidities, individual tolerance, as well as blood pressure and heart rate values. If blood pressure is not controlled according to current guidelines [47], patients are offered additional antihypertensive treatment usually consisting of an ARB. Based on the above evidence [8,11,30], the latter may actually be at least as effective as celiprolol in reducing the rate of vascular events, but further research is needed.

Similar protocols for clinical management are being adopted by other specialized centers across several countries [10,11,28]. Notwithstanding this, there are currently no evidence-based guidelines for management and surveillance [48], and an individualized approach is likely warranted.

## 10. Conclusions

vEDS is a condition with a dramatic impact on affected patients. Although outcomes have significantly improved in recent years, much effort is still needed to fully understand the pathophysiological mechanisms of the disease and to provide effective treatment strategies. Beyond celiprolol, other cardiovascular drugs including ARBs may also contribute in the prevention of vascular events. Although the PREVEnt study was stopped early, inhibitors of the PLC/IP3/PKC/ERK signaling pathway could also play a role in the future. Gene therapy, particularly A-to-I conversion by RNA editing systems, is another promising curative approach in this setting. Lastly, miRNAs involved in vascular ECM structure and turnover could emerge as critical molecular targets for vEDS, providing real hope to affected patients.

## Figures and Tables

**Table 1:** Evidence on different pharmacological strategies in vascular Ehlers–Danlos syndrome from experimental models.

Study	Design	Main Results
Briest et al. (2011) [22]	Animal model *Col3a1* haplo-insufficient mice untreated or treated with doxycycline	Treatment with doxycycline normalized MMP-9 activity, increased collagen content in the tunica media of the abdominal aorta, and reduced stress-induced aortic lesions in *Col3a1* haplo-insufficient mice
Tae et al. (2012) [23]	Animal model *Col3a1* haplo-insufficient mice untreated or treated with doxycycline	Treatment with doxycycline normalized MMP-9 expression, increased collagen content in the tunica media of the aorta, and prevented the development of spontaneous aortic lesions in *Col3a1* haplo-insufficient mice
Dubacher et al. (2020) [21]	Animal model *Col3a1*^m1Lsmi/+^ mice untreated or treated with celiprolol, doxycycline, or losartan	Treatment with celiprolol and doxycycline but not losartan increased the maximum tensile force of the thoracic aortic wall in *Col3a1*^m1Lsmi/+^ mice
Bowen et al. (2020) [26]	Animal model *Col3a1*^G209S/+^ and *Col3a1*^G938D/+^ mice untreated or treated with several therapeutic strategies including celiprolol, ruboxistaurin, cobimetinib, hydralazine, bicalutamide, hydralazine-bicalutamide, or hydralazine-spironolactone	Celiprolol accelerated rather than reduced death from aortic rupture in both models, whereas attenuation of PLC/IP3/PKC/ERK signaling through ruboxistaurin, cobimetinib, or hydralazine significantly reduced the risk of death. Protection with hydralazine was lost at sexual maturity and maintained in case of concomitant administration of androgen receptor antagonists (bicalutamide, spironolactone)
Legrand et al. (2022) [8]	Animal model *Col3a1*^+/G182R^ mice untreated or treated with several therapeutic strategies including celiprolol, propranolol, amlodipine, propranolol-amlodipine, losartan, hydralazine, or celiprolol-hydralazine	No benefit on mortality rate emerged with celiprolol, propranolol, amlodipine, propranolol-amlodipine, hydralazine, or celiprolol-hydralazine vs. untreated *Col3a1*^+/G182R^ mice, with a trend toward a worsening mortality at the age of 15 weeks with amlodipine (*p* = 0.068). Treatment with losartan decreased the 24-week mortality rate (17.6% vs. 62.5%, *p* = 0.021)
Ishikawa et al. (2023) [27]	Case report Patient with heterozygous pathogenic variant (c.1484G>A; p. Gly495Glu) in *COL3A1* undergoing skin biopsy before and after 3 years of celiprolol administration	The expression level of procollagen III did not change, but the size differences in collagen fibrils improved. Furthermore, the ER dilatation observed in skin fibroblasts before treatment, which indicated ER stress was no longer noticed after 3 years of treatment

ER: endoplasmic reticulum; MMP: matrix metalloproteinase.

**Table 2:** Main findings from clinical studies using different pharmacological strategies in vascular Ehlers–Danlos syndrome.

Study	Design	Follow-Up Duration	Main Results
Ong et al. (BBEST study) (2010) [5]	Multicenter, randomized, open trial with blinded assessment of clinical events in eight centers in France and one in Belgium. Patients (n = 53) with clinical vEDS (33 with molecular confirmation) randomly assigned to celiprolol or to no treatment. Primary endpoints: arterial events (rupture or dissection, fatal or not)	47 months (SD 5)	Primary endpoints were reached by five (20%) in the celiprolol group and by 14 (50%) controls (HR 0.36; 95%CI 0.15–0.88; *p* = 0.040). Adverse events were severe fatigue in one patient after starting 100 mg celiprolol and mild fatigue in two patients related to dose uptitration. Brachial SBP and PP increased after celiprolol but not in untreated patients. Carotid Young’s elastic modulus increased while distensibility decreased in the celiprolol group compared with controls
Frank et al. (2019) [10]	Retrospective cohort study on 144 patients with molecularly confirmed vEDS followed in a French referral center between 2000 and 2017 (90.3% on celiprolol) in addition to usual care	5.3 years (IQR 3.2–8.4)	Survival was 80.7% (95%CI 67.8–93.6%) in those treated with celiprolol vs. 48.5% (95%CI 19.7–77.4%) in those not treated (*p* < 0.001) after 11.1 years of follow-up. The best protection was observed at the dose of 400 mg daily vs. lower doses (*p* = 0.003)
Baderkhan et al. (2021) [28]	Retrospective cohort study on 40 patients with molecularly confirmed vEDS followed in a Swedish referral center between 2011 and 2019 (100% on celiprolol)	22 months (range 1–98). Total follow-up 106 patient years	The annual risk of a major vascular event was 4.7%, similar to the treatment arm of the BBEST trial (5%) and lower than in the control arm of the same trial (12%). Fourteen patients experienced one or more side effects: six patients had severe side effects that prevented the target dose from being reached, four terminated the treatment as mentioned above, and four patients continued treatment at target dose, despite side effects
Buso et al. (2023) [9]	Retrospective cohort study on 26 patients with molecularly confirmed vEDS followed in an Italian referral center between 2011 and 2023 (100% on celiprolol)	72 months (range 10–140). Total follow-up 125 patient years	The yearly risk of symptomatic, life-threatening vascular events was 6.4%. The percentages were higher when considering all symptomatic vascular events was (8.8%) and asymptomatic ones (14.4%). Most events occurred after reaching the dose of 400 mg daily. Celiprolol therapy was generally well tolerated, with only one patient reporting fatigue with doses above 200 mg daily

CI: confidence interval; HR: hazard ratio; IQR: interquartile range; SD: standard deviation; vEDS: vascular Ehlers–Danlos syndrome.

## Data Availability

No new data were created or analyzed in this narrative review. Therefore, data sharing is not applicable to this article.
